# Strategies to Modulate the Redifferentiation of Chondrocytes

**DOI:** 10.3389/fbioe.2021.764193

**Published:** 2021-11-22

**Authors:** Xiaoshen Hu, Weiyang Zhang, Xiang Li, Dongling Zhong, Yuxi Li, Juan Li, Rongjiang Jin

**Affiliations:** ^1^ School of Health Preservation and Rehabilitation, Chengdu University of Traditional Chinese Medicine, Chengdu, China; ^2^ Shool of Sports Medicine and Health, Chengdu Sport University, Chengdu, China; ^3^ School of Acupuncture-Moxibustion and Tuina, Chengdu University of Traditional Chinese Medicine, Chengdu, China

**Keywords:** chondrocytes, redifferentiation, dedifferentiation, cartilage tissue engineering, arthropathy

## Abstract

Because of the low self-healing capacity of articular cartilage, cartilage injuries and degenerations triggered by various diseases are almost irreversible. Previous studies have suggested that human chondrocytes cultured *in vitro* tend to dedifferentiate during the cell-amplification phase and lose the physiological properties and functions of the cartilage itself, which is currently a critical limitation in the cultivation of cartilage for tissue engineering. Recently, numerous studies have focused on the modulation of chondrocyte redifferentiation. Researchers discovered the effect of various conditions (extracellular environment, cell sources, growth factors and redifferentiation inducers, and gene silencing and overexpression) on the redifferentiation of chondrocytes during the *in vitro* expansion of cells, and obtained cartilage tissue cultured *in vitro* that exhibited physiological characteristics and functions that were similar to those of human cartilage tissue. Encouragingly, several studies reported positive results regarding the modulation of the redifferentiation of chondrocytes in specific conditions. Here, the various factors and conditions that modulate the redifferentiation of chondrocytes, as well as their limitations and potential applications and challenges are reviewed. We expect to inspire research in the field of cartilage repair toward the future treatment of arthropathy.

## Introduction

A multitude of diseases, such as osteoarthritis and trauma, lead to the degeneration and defect of articular cartilage; because articular cartilage hardly repairs itself, the loss of articular cartilage implies that these patients would have limited mobility and even disability (Smolen et al., 2016; Krishnan and Grodzinsky, 2018). Researchers have attempted to repair cartilage using a variety of techniques. The most recognized among them is autologous chondrocyte implantation (ACI), which uses autologous chondrocytes that are cultured and expanded *in vitro* for repairing cartilage by transplanting the expanded chondrocytes or cartilage-like tissue shaped using tissue-engineering technology into joints. However, one major problem of ACI is that autologous chondrocytes would dedifferentiate during the process of proliferation *in vitro*, become fibrous-like cells, and lose the physiological characteristics of the chondrocytes themselves. Therefore, researchers aim to develop methods to solve this problem and have assessed a variety of factors and techniques that may regulate the redifferentiation of chondrocytes *in vitro*, including the extracellular environment, cell sources, growth factors and redifferentiation inducers in the cell culture environment, and gene silencing and overexpression. In this study, we summarized and analyzed relevant studies in this field to provide strategies for the modulation of chondrocyte redifferentiation.

## Cell Sources

The main cell source of ACI is a cartilage sample from the low-weight-bearing area of the patient’s sick joint (mainly the knee joint), which provides chondrocytes for cultivation and expansion *in vitro*, followed by their implantation into the articular cartilage defect (Jones and Peterson, 2006; Minas et al., 2014; Mistry et al., 2017). Current research shows that chondrocytes extracted from articular hyaline cartilage remain more effective in repairing cartilage than human bone marrow stem cells (HBMSCs), even though the former usually undergo dedifferentiation during *in vitro* culture and expansion (Makris et al., 2015). The implantation of HBMSCs into the defect of articular cartilage usually causes the formation of fibrocartilage, which is not sufficient for joints that have weight-bearing functions.

In turn, the use of human nasal septal chondrocytes in ACI has better clinical application potential (Mumme et al., 2016; von Bomhard et al., 2017). Compared with articular cartilage cells, human nasal septum chondrocytes exhibit a superior ability to reproducibly generate hyaline-like cartilage tissues, based on their plasticity of adaption to a joint environment. Moreover, polydactyly cartilage derived from patients with polydactyly in infancy may be another option in this setting. Polydactyly chondrocytes also have the potential for use in stable cartilage production (Cavalli et al., 2019). Similarly, microtia chondrocytes from patients with microtia are another alternative cell source, as dedifferentiated microtia chondrocytes transform into redifferentiated microtia chondrocytes after culturing in a three-dimensional (3D) chondrogenic culture system (He et al., 2020). Relevant researches on cell source have been summarized in Table 1.

**TABLE 1:** Cell source modulates the redifferentiation of chondrocytes.

Aspects	Factors	Modulation of chondrocyte redifferentiation	References
Cell source	Human knee chondrocytes	Commonly used cell sources	Jones and Peterson, (2006); Minas et al., (2014); Mistry et al., (2017)
	Human nasal septal chondrocytes	Better reproducible ability to generate hyaline-like cartilage tissue	Mumme et al., (2016); von Bomhard et al., (2017)
	Human polydactyly chondrocytes	Potential for stable cartilage production	Cavalli et al. (2019)
	Human microtia chondrocytes	3D chondrogenic culture system is needed	He et al. (2020)

For practical application, it is feasible to use cartilage tissue from the patient’s own diseased joint as a cell source for ACI. However, the state of articular cartilage cells in different patients is not the same. Therefore, the reproducibility of the results of this approach in different studies is questionable.

## Extracellular Microenvironment

During the process of *in vitro* expansion of chondrocytes, a variety of extracellular microenvironmental conditions may affect their dedifferentiation and redifferentiation, including the culture temperature, hypoxia, 3D culture, extracellular matrix (ECM), and hydrogel. Several studies have confirmed that hypoxia and 3D culture are beneficial for the redifferentiation of chondrocytes. Because 3D culture lacks uniform standards, the quality of studies of 3D culture is inconsistent. In turn, the fact that a low temperature (32.2°C) delays the dedifferentiation of chondrocytes may be because it attenuates all biological processes in chondrocytes. Finally, hydrogels have great potential for the redifferentiation of chondrocytes; however, the related parameter standard of hydrogels requires further investigation.

### Culture Temperature

In 3D pellet culture, a temperature of 37°C promotes chondrocyte redifferentiation to a greater extent than does a temperature of 32.2°C. Moreover, a temperature of 32.2°C slows down the proliferation rate of chondrocytes significantly. In monolayer culture, hypothermia at 32.2°C retarded the dedifferentiation and proliferation rate of chondrocytes significantly (von Bomhard et al., 2017). Hypothermia has the potential to avoid dedifferentiation in monolayer culture. Another study demonstrated that a culture temperature of 41°C inhibited the redifferentiation of chondrocytes and the formation of ECM compared with 37°C (Ito et al., 2015).

### Hypoxia

The environment in human healthy joints is hypoxic with negative pressure (Stegen et al., 2019). Does hypoxia regulate the redifferentiation of chondrocytes? Many studies (Duval et al., 2009; Markway et al., 2013; Das et al., 2015; Ollitrault et al., 2015; Rakic et al., 2017; Öztürk et al., 2017; Jeyakumar et al., 2019) have investigated the effect of hypoxic conditions on the redifferentiation of chondrocytes during *in vitro* expansion, with the results basically indicating that hypoxia has a positive effect on the redifferentiation of chondrocytes. In addition, several studies have reported that a partial oxygen pressure (pO₂) of 2.5% achieves the best effect in improving the expression of chondrocytic markers (Acan and Col2a1) and suppressing the expression of dedifferentiation markers (Col1a1 and Col3a1) (Jahr et al., 2019).

### Three-Dimensional Culture

**FIGURE 1:**
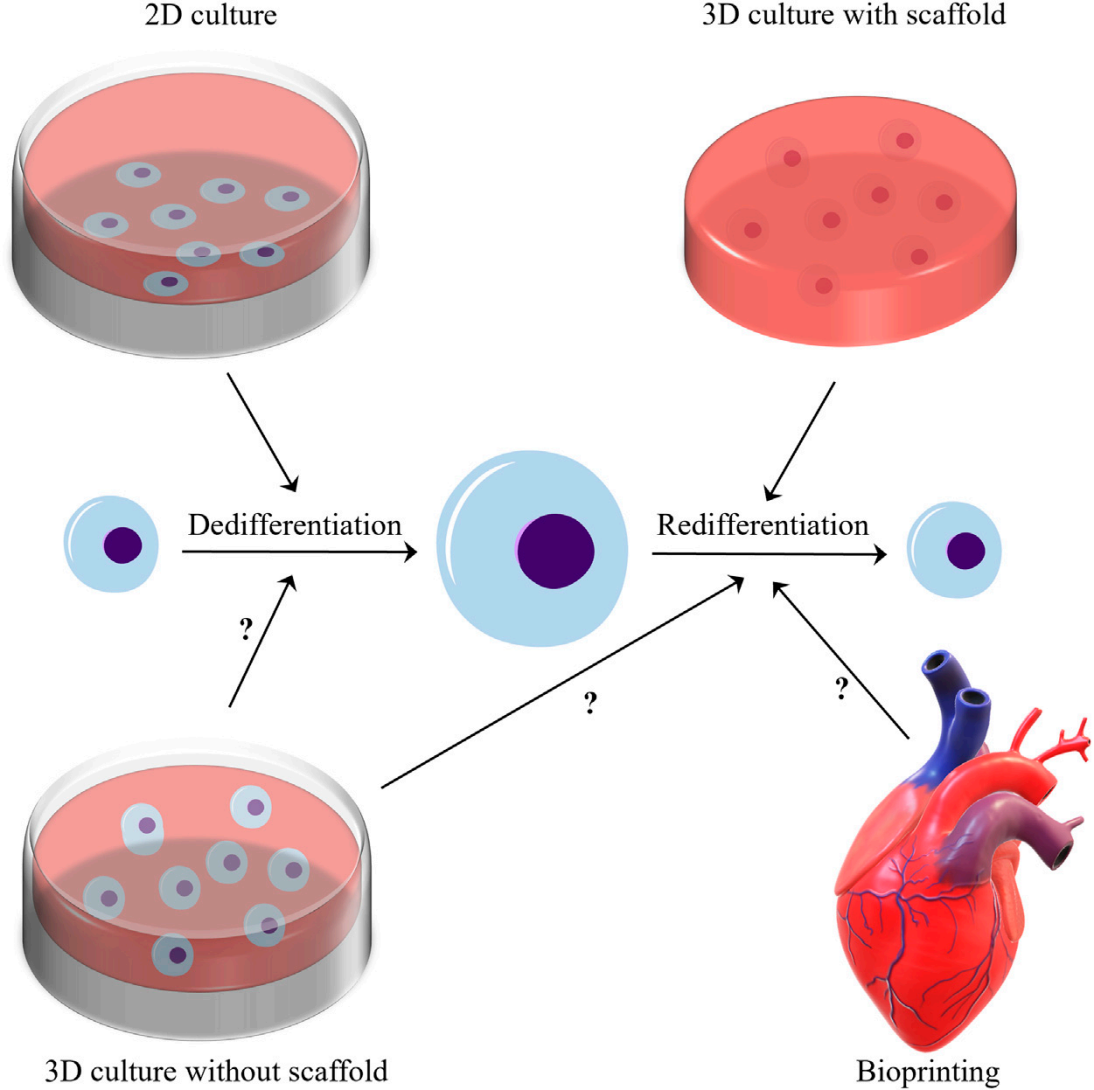
In the microenvironment of two-dimensional culture, chondrocytes generally undergo dedifferentiation after proliferation. Three-dimensional culture with scaffolds has a good ability to modulate the redifferentiation of chondrocytes. And does the three-position culture without scaffold and bioprinting have the ability to modulate the redifferentiation of chondrocytes? This issue needs further research to clarify.

At present, 3D culture is widely used in cell culture, because researchers increasingly find that the extracellular environment of 2D culture is far from the real extracellular microenvironment. 3D culture can be classified into 3D culture methods with and without scaffolds (Wu X. et al., 2021), and Three-dimensional culture with scaffolds is widely used to redifferentiate chondrocytes (Caron et al., 2012). This type of culture is a common approach that is used to induce and maintain chondrocyte redifferentiation, and generate ECM. Numerous studies (von Bomhard et al., 2017; He et al., 2020; Caron et al., 2012; Takahashi et al., 2007; Schulze-Tanzil, 2009; Mukaida et al., 2005; Ahmed et al., 2014) have shown that 3D culture with scaffolds can regulate the redifferentiation of chondrocytes. Perhaps because researchers generally believe that 3D culture with scaffolds is closer to mammalian cartilage in physical properties, almost all 3D cultures with scaffolds are used to regulate chondrocyte redifferentiation. But can a scaffold-free 3D culture system regulate the redifferentiation of chondrocytes? This is a question worth studying. The application of bioprinting in cartilage tissue engineering has made great progress, but its effect on the redifferentiation of chondrocytes needs further research (Han et al., 2021). Besides, there is no criterion for 3D culture, which hinders its application to the induction of the redifferentiation of chondrocytes (represented in Figure 1).

### Extracellular Matrix and Hydrogels

It is believed that 3D culture can induce chondrocyte differentiation. Researchers have explored many bionic materials in this context. Because of the wide range of feasible properties and the ability to culture cells in material, hydrogels have become a promising extracellular scaffold (Vega et al., 2017). Based on their ability to adjust their elasticity, hydrogels are an option to regulate the redifferentiation of chondrocytes (Bachmann et al., 2020). Fibrin hydrogels with elasticity close to 30 kPa have a shape that is similar to that of the natural physiological cartilage tissue and is able to induce the synthesis of physiological ECM constituents, such as glycosaminoglycans (sGAG) and collagen type II (Bachmann et al., 2020). In addition, the adhesion-site density of the hydrogels has been proven to affect the redifferentiation of chondrocytes, whereas the hardness of the hydrogel substrate does not (Schuh et al., 2012). Researchers have discovered several novel types of hydrogel for the proliferation of chondrocytes *in vitro*. For example, a microcavitary alginate hydrogel can help dedifferentiated chondrocytes to redifferentiate and recover the capability of synthesizing ECM (Zeng et al., 2015). Gelatin–methacryloyl hydrogels, tyrosinase-crosslinked alginate sulfate tyramine hydrogels, and composite microfibers also exhibit potential for the engineering of cartilage-like tissues for 3D cultured chondrocytes (Klotz et al., 2016; Angelozzi et al., 2017; Öztürk et al., 2020). Self-assembled dendritic DNA hydrogel possesses inherent biocompatibility, biodegradability, and unique programmability, and has shown great potential in three-dimensional cell culture (Wu J. et al., 2021), but its effect on the redifferentiation of chondrocytes needs further investigation.

Compared with hydrogels, the decellularized matrix (DCM) derived from natural tissues is more suitable for cell culture and is closer to the physiological environment; however, the DCM has the disadvantage of being arduous to generate in large quantities. The mesenchymal stem cell (MSC)-derived extracellular matrix (MSC-ECM) is a natural biological material with strong bioactivity and excellent biocompatibility. Studies have shown that MSC-ECM derived from decellularized human bone marrow is an excellent culture substrate for the expansion of chondrocytes, with firm cartilage formation being observed after 21 days of culture *in vitro* (Yang et al., 2018). The meniscus-derived DCM has shown the promising effect of promoting the proliferation of chondrocytes. With the intervention of combined growth factors, meniscus-derived DCM can modulate the differentiation of chondrocytes (Liang et al., 2020). In addition, ECM deposited by synovial-derived stem cells significantly enhanced the proliferation of chondrocytes and retarded the dedifferentiation of the proliferated chondrocytes (Pei and He, 2012).

### Growth Factors and Redifferentiation Inducers

Signal transduction mediated by the TGFβ superfamily plays an important role in the regulation of cell growth, differentiation, and development in many biological systems. The TGFβ and bone morphogenetic proteins (BMP) signaling pathways are regulated at multiple levels by the MAPK signaling pathway. In addition, in some cases, the TGFβ signaling pathway can also affect Smad-independent signal pathways, including the Erk, SAPK/JNK, and p38 MAPK signal pathways. Therefore, there exists cross-talk among the members of the TGF-β superfamily to regulate the redifferentiation of chondrocytes. Some studies have demonstrated the positive effect of the combined application of growth factors on the regulation of redifferentiation, but it is difficult to ignore the negative effect of some growth factors on the repair of cartilage, such as BMP2 can induce endochondral solidification (Zhou et al., 2016) and BMP7 may induce heterotopic ossification (Spiro et al., 2010); therefore, additional research is needed to assess the combinatorial effect of different growth factors.

#### Transforming Growth Factor β Family

Growth factors play a crucial role in the processes of cell proliferation, extracellular matrix synthesis, phenotype maintenance, induction of dedifferentiated chondrocytes or MSCs, and cartilage formation. The members of the transforming growth factor β (TGFβ) family are particularly paramount for these roles (Freyria and Mallein-Gerin, 2012; Dexheimer et al., 2016). Members of the TGFβ family include TGFβs, BMPs, and growth and differentiation factors (GDFs) (Thielen et al., 2019). The TGFβ family regulates cell-fate decisions during development, and tissue homeostasis and regeneration. The members of the TGFβ family are major participants in cartilage and bone formation, tumorigenesis, fibrotic diseases, immune dysfunction, and various congenital diseases (Wozney et al., 1988; David and Massagué, 2018).

Humans express three types of TGFβ, including TGFβ1, TGFβ2, and TGFβ3 (Chen and Ten Dijke, 2016). TGFβs are secreted by chondrocytes and combine with their ECM. Moreover, the TGFβ signaling pathways are related to the production and maintenance of cartilage ECM (van der Kraan et al., 1992; Jahr et al., 2019; Bachmann et al., 2020) and have a positive antiinflammatory effect in cartilage (Rédini et al., 1993; Takahashi et al., 2005). Of note, the regulation of chondrocyte hypertrophy is a particularly crucial role of TGFβ signaling pathways. The signaling transduction of pSMAD2/3 induced by TGFβs blocks the hypertrophy and terminal differentiation of chondrocytes (Yang et al., 2001; Li et al., 2006; Blaney Davidson et al., 2009; Kim et al., 2012). In some studies, TGFβs are used as a supplement to stimulate the redifferentiation of cultured chondrocytes *in vitro* (Jakob et al., 2001; Lee et al., 2005; van der Windt et al., 2010; Baugé et al., 2013; Bianchi et al., 2017; Bianchi et al., 2019). In contrast, the inhibition of the activity of TGFβ1 during cell expansion *in vitro* increases the redifferentiation ability of chondrocytes and inhibits their hypertrophy (Narcisi et al., 2012). TGFβ signaling transduction is pivotal in the regulation of the dedifferentiation and redifferentiation of chondrocytes (Dong et al., 2005). Nevertheless, this is a complex process, and additional research is necessary to clarify this issue.

In humans, the BMP signaling pathways include more than 20 distinct ligands, four type I receptors (Bmpr1a, Bmpr1b, Acvr1, and Alk1) and three type II receptors (Bmpr2, Acvr2a, and Acvr2b) (Antebi et al., 2017). These components can be combined with each other to assemble hundreds of different receptor–ligand complexes. Each complex is composed of two type I receptors and two type II receptor-binding ligands. The current research shows that the BMP pathway usually operates through multiple ligands and receptors (Lorda-Diez et al., 2014; Salazar et al., 2016). For example, BMP9 and BMP10 jointly regulate the production of vasculature (Ricard et al., 2012; Chen et al., 2013), and the existence of heterodimers of BMP2/6, BMP2/7, and BMP4/7 has been confirmed *in vivo* and *in vitro* (Bragdon et al., 2011).

BMP2 is a recognized chondrocyte maturation and hypertrophy inducer, and is able to induce cartilage differentiation, osteogenic differentiation, and endochondral ossification of stem cells (Zhou et al., 2016). BMP2’s regulation of chondrocyte redifferentiation is controversial in different studies. Davidson observed a lack of articular cartilage hypertrophy in mice overexpressing BMP2 for 6 weeks (Blaney Davidson et al., 2015). In addition, overexpression of BMP2 does not have a significant effect on articular cartilage damage, but it induces extensive osteophyte hyperplasia (Blaney Davidson et al., 2015). Rakic and colleagues combined BMP2 with a Col1a1 small interfering RNA (siRNA) and 3D hypoxia cell culture to induce chondrocyte redifferentiation successfully (Rakic et al., 2017). It is recognized that the transcription factor RUNX2 can induce bone formation, but the induction into cartilage requires the combined application of transcription factors SOX9 and RUNX2 (Eames et al., 2004). Perhaps similar to the transcription factor RUNX2’s role in cartilage formation, BMP2 may be necessary to regulate the redifferentiation of chondrocytes, and the use of BMP2 alone to induce chondrocyte redifferentiation is obviously a wrong method. It is known that BMPs and TGFβs are usually combined to participate in the proliferation and differentiation of chondrocytes (Miyamoto et al., 2007; Shintani et al., 2013). The growth factor mixture is a novel approach that is used to modulate the redifferentiation of chondrocytes. A mixture containing TGFβ1, BMP2, GDF5, BMP6, and fibroblast growth factor 2 (FGF2) has the potential to stably drive cartilage formation (Mendes et al., 2018). Similarly, TGF-β1, GDF-5, and BMP-2 as a combined inducer can significantly stimulate the expression of cell cartilage genes and secrete collagen type II (Murphy et al., 2015).

BMP7 promotes the expression of ECM in cartilage. Previous studies have suggested that BMP7 induces heterotopic ossification (Spiro et al., 2010); however, unlike BMP3, BMP7 does not induce excessive osteophyte proliferation (Hunter et al., 2010). The combination therapy of BMP7 and TGFβ3 has the advantage to stimulate the redifferentiation of chondrocytes (Huang et al., 2018). In addition, a scaffold composition was able to continuously release BMP7 and TGFβ3 and promote chondrocyte differentiation (Crecente-Campo et al., 2017). This scaffold can encapsulate a combination of different growth factors and exert a synergistic effect between them, to regulate chondrocyte redifferentiation. An interesting study demonstrated that, after 4 weeks of injection of human synovial MSCs overexpressing BMP7 into the thighs of mice, researchers discovered an implant that histologically resembled a rudimentary joint (Roelofs et al., 2017), in which there was articular cartilage, subchondral bone containing marrow, and a growth plate. This phenomenon warrants further investigation.

The role of BMP4 and BMP6 in the regulation of chondrocyte redifferentiation is poorly understood. BMP4 promotes the secretion of chondrocyte proteoglycan and the expression of collagen type II (Luyten et al., 1994). Similarly, BMP6 promotes the expression of proteoglycan in human cartilage (Bobacz et al., 2003; Chubinskaya et al., 2008). However, a hypertrophic effect of BMP6 has been observed in ATDC5 cells (Ito et al., 1999).

#### Other Growth Factors

FGF2 is a growth factor with a wide range of mitogenic and cell-survival activities (Nawrocka et al., 2020). Adding FGF2 to chondrocytes cultured *in vitro* promotes cell proliferation, induces rapid and reversible cell dedifferentiation, and leads to the expression of cartilage marker genes and secretion of ECM after inducing redifferentiation (Jakob et al., 2001; Claus et al., 2012). Several studies have used FGF2 as an inducer to promote the expansion of chondrocytes *in vitro* (Hendriks et al., 2006; Lee et al., 2017; Endo et al., 2019; Kikuchi and Shimizu, 2020; Song et al., 2021). After a large number of dedifferentiated chondrocytes was obtained, other inducers were used to induce the redifferentiation of chondrocytes (Martin et al., 2001; Dufour et al., 2019). Similarly, hypoxia combined with FGF2 to induce *in vitro* culture may be a better method for the expansion of chondrocytes *in vitro*. Moreover, hypoxia combined with FGF2 may improve the growth rate of cells, reduce the level of dedifferentiation during expansion, and have a greater ability to induce redifferentiation (Koh et al., 2017).

The insulin-like growth factor 1 (IGF1) and its downstream pathway play a major role in normal growth and aging, whereas the levels of serum IGF1 decrease with age (Frater et al., 2018). Similar to the role of FGF2, the activation of the IGF1 pathway could drive the rapid proliferation and hypertrophy of chondrocytes (Yakar et al., 2018). Several studies have also confirmed that IGF1 is not conducive to the redifferentiation of chondrocytes (Frerker et al., 2021). However, the combined application of IGF1 and TGFβs has been successful in regulating the redifferentiation of chondrocytes (Witt et al., 2017; Klinder et al., 2020).

**FIGURE 2:**
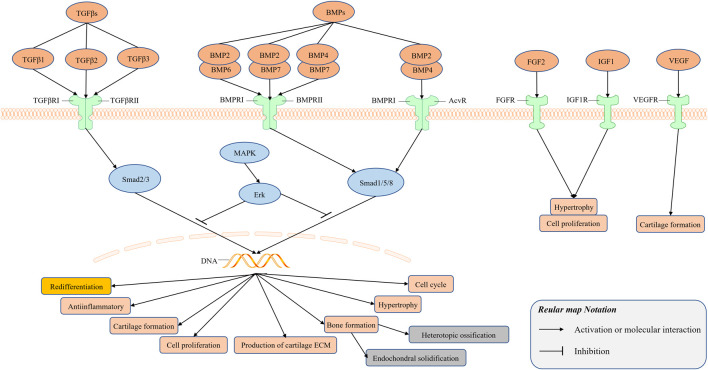
Signal transduction mediated by growth factors plays an important role in the regulation of cell growth, differentiation and development in many biological systems. There exists cross-talk among the members of the TGF-β superfamily to regulate the redifferentiation of chondrocytes. TGFβs bind to its receptor TGFβ receptor Ⅰ (TGFβRⅠ) and Ⅱ (TGFβRⅡ) to form a complex, and BMPs bind to its receptor BMP receptor Ⅰ (BMPRⅠ) and Ⅱ (BMPRⅡ), Acv receptor (AcvR) to form a complex.

The vascular endothelial growth factor (VEGF) is involved in angiogenesis and the negative regulation of cartilage growth by stimulating vascular invasion and ossification (Apte et al., 2019). Inhibition of VEGF function has a positive effect on cartilage formation by human-derived nasal chondrocytes (Carlevaro et al., 2000; Medeiros Da Cunha et al., 2017), and the obstruction of vascular invasion during bone healing, rather than osteogenic differentiation, is beneficial to cartilage formation by bone progenitor cells (van Gastel et al., 2020). The relevant researches of growth factors have been summarized and illustrated in Figure 2.

#### Other Redifferentiation Inducers

Platelet derivatives (such as platelet-rich plasma (PRP), hyperacute serum (HAS), platelet lysate (PL), and the <5 kDa fraction of human serum albumin (LMWF5A)) are used to overcome the dedifferentiation caused by the *in vitro* expansion used to achieve a sufficient number of cells and variable oxygen tension. Recent studies have confirmed that HAS enhances the proliferation of chondrocytes and PRP boosts the proliferation and redifferentiation of dedifferentiated chondrocytes (Jeyakumar et al., 2017; Jeyakumar et al., 2019). The current research on PL supports its application to promote the expansion of chondrocytes; however, there are doubts regarding the regulation of chondrocyte redifferentiation (Rikkers et al., 2020; Liau et al., 2021). Recent studies of LMWF5A have shown its potential in inducing fibroblast-like chondrocytes to redifferentiate into functional chondrocytes (Hausburg et al., 2018).

Similarly, inhibitors of several important pathways involved in the regulation of chondrocyte dedifferentiation (such as the MEK-ERK1/2 and WNT signaling pathways) are used to explore their role in the regulation of chondrocyte redifferentiation. The expression of ERK1/2 increases during the process of chondrocyte dedifferentiation, whereas it decreases during the process of redifferentiation. Activation of the MEK-ERK1/2 pathway causes the dedifferentiation of chondrocytes (Provot et al., 2008). PD0325901, which is an inhibitor of ERK1/2, reverses the dedifferentiation and leads to redifferentiation of chondrocytes (Wang et al., 2018). Inhibitors of the WNT signaling pathway, Dickkopf 1 homolog (DKK1), and frizzled-related protein (FRZB), have been shown to be essential for the early steps of chondrocyte differentiation (Zhong et al., 2016). They are necessary for the promotion of the redifferentiation of articular chondrocytes and the inhibition of their hypertrophy and differentiation. Moreover, the activity of calcineurin (Cn) in human articular chondrocytes is significantly increased during dedifferentiation. Therefore, studies of FK506, an inhibitor of Cn, confirmed that it could promote the expression of the chondrogenesis markers collagen type Ⅱ, proteoglycan, and SOX9 in expanded chondrocytes (van der Windt et al., 2010).

Moreover, the natural plant compound oleuropein is a polyphenol extracted from the leaves and fruits of olives that has been verified to induce the redifferentiation of chondrocytes in patients with osteoarthritis (OA) and to reduce the number of senescent cells in joint tissues (Varela-Eirín et al., 2020). The relevant researches on the extracellular environment have been summarized in Table 2.

**TABLE 2:** Various factors in the extracellular microenvironment modulate the redifferentiation of chondrocytes.

Aspects	Factors	Modulation of chondrocyte redifferentiation	References
Extracellular microenvironment	Culture temperature	Culture temperature of 37°C promotes chondrocyte redifferentiation	von Bomhard et al., (2017); Ito et al., (2015)
	Hypoxia	Hypoxia has a positive effect on the redifferentiation of chondrocytes	Duval et al., (2009); Markway et al., (2013); Das et al., (2015); Ollitrault et al., (2015); Rakic et al., (2017); Öztürk et al., (2017); Jeyakumar et al., (2019)
	Three-dimensional culture	3D culture has a positive effect on the redifferentiation of chondrocytes	von Bomhard et al., (2017); He et al., (2020); Caron et al., (2012); Takahashi et al., (2007); Schulze-Tanzil, (2009); Mukaida et al., (2005); Ahmed et al., (2014)
	Hydrogels	The elasticity of hydrogels, the density of the adhesion sites, and their configuration methods will all mediate the redifferentiation of chondrocytes; however, there is currently a lack of standardized methods for evaluating hydrogels	Schuh et al., (2012); Zeng et al., (2015); Klotz et al., (2016); Angelozzi et al., (2017); Bachmann et al., (2020); Öztürk et al., (2020)
	ECM	DCM has a positive effect on the redifferentiation of chondrocytes, with the disadvantage that it is difficult to produce in large quantities	Pei and He, (2012); Yang et al., (2018); Liang et al., (2020)
	Transforming growth factor β family	The effect of a single TGFβ as an inducer is limited, and the combined application of multiple TGFβ warrants further research	Jakob et al., (2001); Dong et al., (2005); Lee et al., (2005); van der Windt et al., (2010); Narcisi et al., (2012); Baugé et al., (2013); Blaney Davidson et al., (2015); Bianchi et al., (2017); Crecente-Campo et al., (2017); Roelofs et al., (2017); Mendes et al., (2018); Bianchi et al., (2019)
	Other growth factors	FGF2 and IGF1 induce cell proliferation and dedifferentiation and, combined with other inducers or hypoxia, can regulate the redifferentiation of chondrocytes	Martin et al., (2001); Hendriks et al., (2006); Lee et al., (2017); Witt et al., (2017); Dufour et al., (2019); Endo et al., (2019); Kikuchi and Shimizu, (2020); Klinder et al., (2020); Song et al., (2021)
	Other redifferentiation inducers	PRP, LMWF5A, and ERK1/2 inhibitors; WNT inhibitors; Cn inhibitors and oleuropein all show potential to modulate the redifferentiation of chondrocytes	van der Windt et al., (2010); Zhong et al., (2016); Jeyakumar et al., (2017); Hausburg et al., (2018); Wang et al., (2018); Jeyakumar et al., (2019); Varela-Eirín et al., (2020)

## Gene Silencing and Overexpression

**FIGURE 3:**
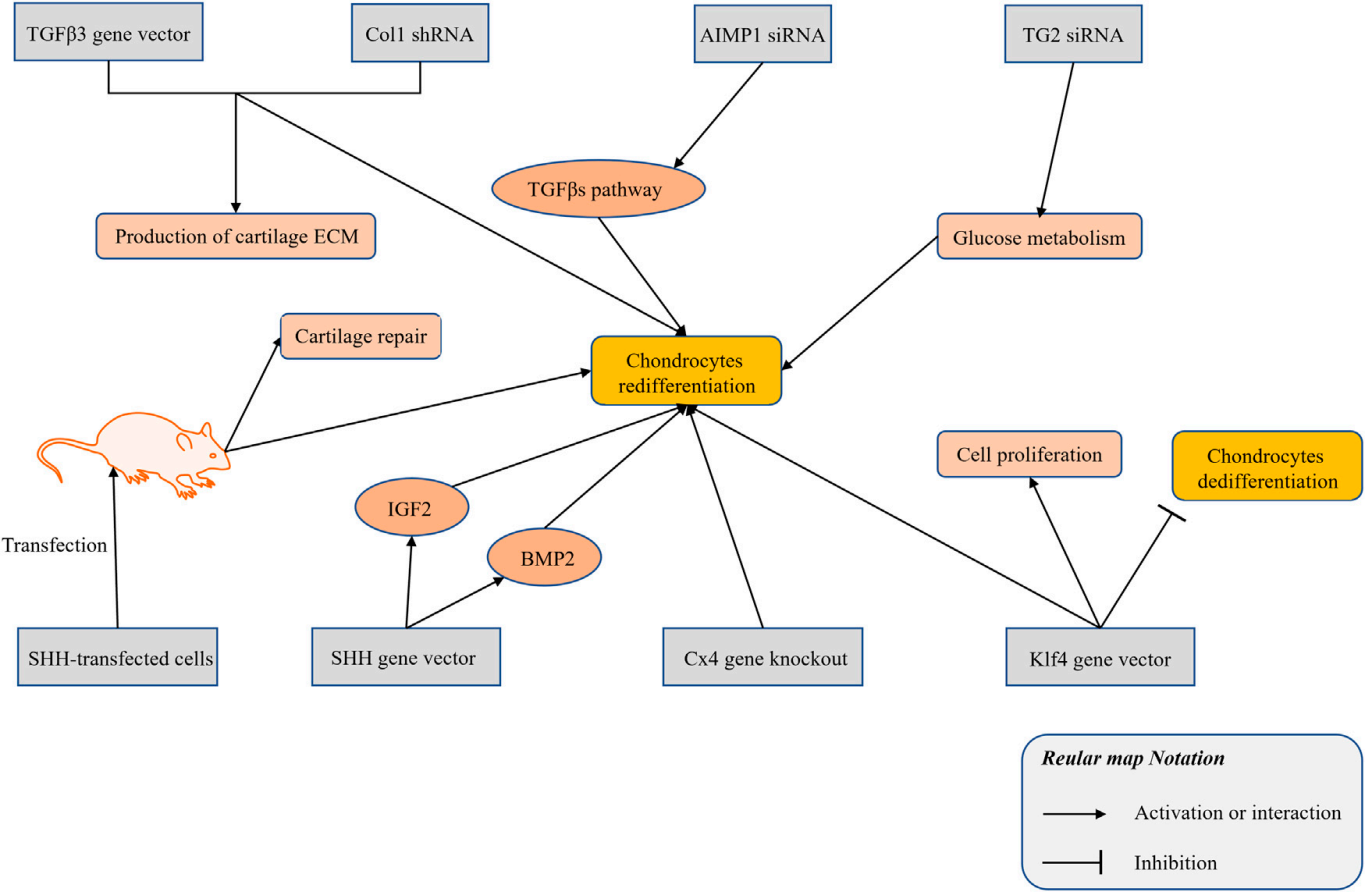
The research progress of gene silencing and expression technology in modulating the chondrocytes redifferentiation.

At present, vectors for the overexpression of genes and interfering RNAs have been used in studies of the redifferentiation of chondrocytes. The combined application of a TGFβ3 adenovirus vector and a Col1 short-hairpin RNA (shRNA) promoted the expression of cartilage marker genes in dedifferentiated chondrocytes, such as collagen type II and proteoglycans (Zhang et al., 2011). The siRNA of aminoacyl-tRNA synthetase-interacting multifunctional protein 1 (AIMP1) restored the TGFβ signaling pathway in degenerated chondrocytes, thereby enhancing the chondrogenic potential of dedifferentiated chondrocytes (Ahn et al., 2016). Transglutaminase 2 (TG2) is increased in chondrocytes in a passage-dependent manner, and enhances cell dedifferentiation (Eckert et al., 2014). Using TG2 siRNA could lead to the redifferentiation of dedifferentiated chondrocytes by enhancing the glucose metabolism process (Ko et al., 2017). Sonic hedgehog (SHH) is involved in the induction of the early chondrogenic differentiation process of limb mesenchymal cells. The use of an *SHH* gene plasmid vector to overexpress dedifferentiated rat chondrocytes increases the expression of SHH and the synthesis of a variety of cell growth factors (e.g., BMP2 and IGF1). Rats transplanted with SHH-transfected cells show better cartilage repair and induction of the redifferentiation of dedifferentiated chondrocytes (Lin et al., 2014). Connexin43 (Cx43) has been validated as a regulator of the transformation between chondrocytes and mesenchymal cells. Downregulation of Cx43 by CRISPR/Cas9 triggers the redifferentiation of OA chondrocytes (Varela-Eirín et al., 2018). The Kruppel-like factor 4 (Klf4) is a multifunctional transcription factor that regulates diverse cellular processes, such as cell growth, proliferation, and differentiation (Ghaleb and Yang, 2017). The *Klf4* gene vector promotes the proliferation and redifferentiation of chondrocytes and inhibits their dedifferentiation (Gurusinghe et al., 2019). The relevant research on the gene expression has been summarized and illustrated in Figure 3.

However, because of ethical issues, the clinical application of gene editing technology remains controversial. Moreover, gene silencing and overexpression do not yield a better effect than do growth factors in regulating the redifferentiation of chondrocytes.

## Summary and Prospect

Articular cartilage is indispensable in the life of all vertebrates. It acts as a buffer against external forces when humans and animals perform various activities. Excessive activity or trauma can cause damage to the articular cartilage, which can lead to restricted movement of the injured joint. Therefore, it is crucial to repair cartilage to restore joint mobility. Cartilage can hardly repair itself, and researchers generally believe that it is due to its extremely poor blood supply and relatively closed intra-articular environment. Researchers had tried to utilize implanting autologous chondrocytes to repair cartilage (Brittberg et al., 1994), but there was no significant effect. Therefore, currently the main clinical treatment for patients with various cartilage injuries suffering from restricted mobility and life disorders is joint replacement surgery. However, the surgical indications for joint replacement surgery determine the limitations of the applicable patients, which can only be used in elderly patients. people. Therefore, repairing cartilage is still the main goal of researchers.

**FIGURE 4:**
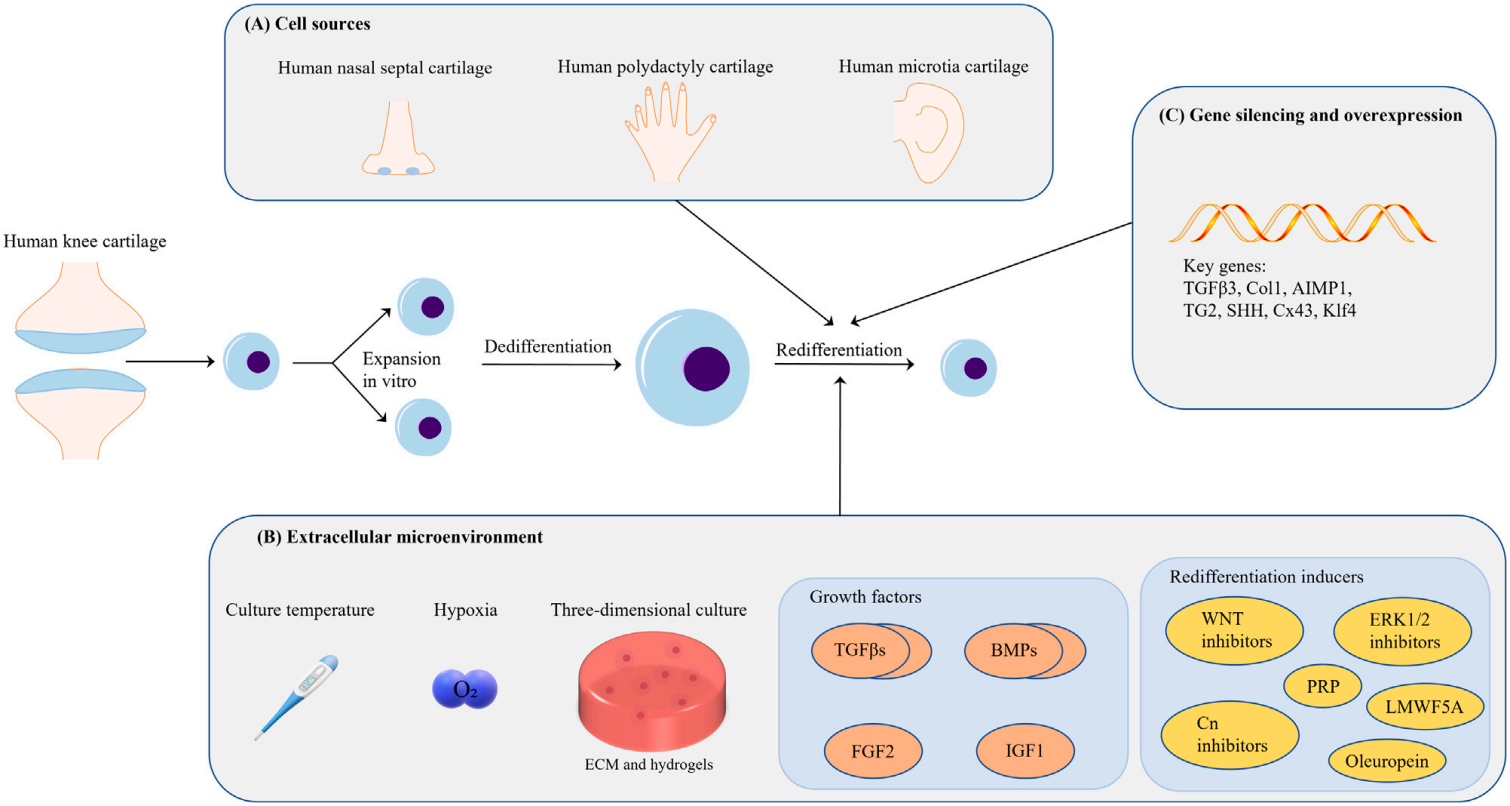
**(A)** In terms of cell source, it is feasible to use the cartilage tissue from the patient’s own diseased joint as a cell source for ACI; chondrogenic diseases such as polydactyly are another potential cell source. **(B)** Many extracellular environmental factors affect cell redifferentiation; however, proper hypoxia and 3D culture are beneficial for chondrocyte redifferentiation. Hydrogels are the medium used for 3D cell culture, but the related parameter standard of hydrogels warrants further investigation. Members of the TGF-β superfamily are currently the most important chondrocyte redifferentiation inducers. The combined application of growth factors may be a good choice in this context. **(C)** Research on gene silencing and overexpression for the regulation of chondrocyte redifferentiation is still focused on growth-factor-related genes; thus, additional investigation is needed in this area.

At present, the technical limitation of ACI lies in the low proliferation ability of autologous chondrocytes. After proliferation, chondrocytes will dedifferentiate and lose their cell function, and their ability to synthesize ECM is low. The author believes that the problem is that the extracellular microenvironment for autologous chondrocyte expansion *in vitro* is very different from the real human *in vivo* environment. Regardless of the two-dimensional culture, culture medium, extracellular matrix and various extracellular mechanical stimuli are different. Therefore, simulating the extracellular microenvironment of chondrocytes for differentiation and redifferentiation as much as possible may be the fundamental method to solve the technical problems of autologous cartilage implantation. Obviously, this is very difficult in terms of current biological technology. This article summarizes various conditions (cell sources, extracellular environment, growth factors and redifferentiation inducers, and gene silencing and overexpression) on the redifferentiation of chondrocytes, hoping to show some surprising progress in this field, and propose a preliminary Strategy (represented in Figure 4).

In summary, the redifferentiation of chondrocytes is modulated at multiple levels. Despite significant progress in this field, many questions remain to be resolved. Regarding the extracellular microenvironment, hypoxia and 3D culture have shown potential to modulate the redifferentiation of chondrocytes. Although the culture temperature, ECM, and hydrogel all have the effect of modulating the redifferentiation of chondrocytes, they all have their own limitations. For example, a high or low temperature is not conducive to cell expansion, and the amount of naturally generated ECM is limited. There is no doubt that the parameters of the hydrogel also need standardized evaluation criteria. TGFβ family members are the most used redifferentiation inducers currently. However, they involve complex biological pathways, which renders it difficult to understand the biological effects of a single member of the TGFβ family. The combined application of different family members may be an appropriate method.

## Conclusion

In conclusion, the strategy of regulating the redifferentiation of chondrocytes is not static, it needs to be adjusted according to factors such as cell source and cell stage. The ideal strategy for modulating chondrocytes redifferentiation may have 3D culture, hypoxia, appropriate temperature, hydrogel or ECM, inducers suitable for cell expansion phase and cell redifferentiation phase after expansion, respectively. As we continue to learn more about these factors in the future, it will be very important to capitalize on these discoveries by modulating redifferentiation of chondrocytes for the treatment of cartilage damage related diseases.
